# Author Correction: Birth of protein folds and functions in the virome

**DOI:** 10.1038/s41586-024-08068-7

**Published:** 2024-09-23

**Authors:** Jason Nomburg, Erin E. Doherty, Nathan Price, Daniel Bellieny-Rabelo, Yong K. Zhu, Jennifer A. Doudna

**Affiliations:** 1 Gladstone–UCSF Institute of Data Science and Biotechnology, San Francisco, CA USA; 2 Department of Molecular and Cell Biology, University of California, Berkeley, Berkeley, CA USA; 3 Innovative Genomics Institute, University of California, Berkeley, Berkeley, CA USA; 4 California Institute for Quantitative Biosciences, University of California, Berkeley, Berkeley, CA USA; 5 Howard Hughes Medical Institute, University of California, Berkeley, Berkeley, CA USA; 6 Molecular Biophysics and Integrated Bioimaging Division, Lawrence Berkeley National Laboratory, Berkeley, CA USA; 7 Department of Chemistry, University of California, Berkeley, Berkeley, CA USA

**Keywords:** Virology, Functional clustering

Correction to: *Nature* 10.1038/s41586-024-07809-y Published online 26 August 2024

In the version of the article initially published, in the “Similarity to non-viral proteins” section, the sentence originally reading “…the AlphaFold database, which contains more than 300,000 proteins from 21 organisms” has now been corrected to “the AlphaFold database, which contains more than 500,000 proteins from 48 organisms”. Additionally, in the Methods, in the “Structural alignments against the AlphaFold databases” section, the text “(downloadable via the Foldseek command ‘foldseek databases Alphafold/Proteome afdb tmp’)” has now been added. These corrections have been made to the HTML and PDF versions of the article.

