# Supplementary material for: Birth of protein folds and functions in the virome
Source: Nature. 2024 Aug 26;633(8030):710–7. doi: 10.1038/s41586-024-07809-y (PMC11410667; doi:10.1038/s41586-024-07809-y)
Supplement: Supplementary file 1 — This file contains uncropped blot images. [file 41586_2024_7809_MOESM1_ESM.pdf]

nature portfolio

https://doi.org/10.1038/s41586-024-07809-y

Supplementary information

Birth of protein folds and functions in the virome

In the format provided by the authors and unedited

Birth of protein folds and functions in the virome

Jason Nomburg[1,2,3], Erin E. Doherty[3,4], Nathan Price[1,2,3], Daniel Bellieny-Rabelo[3,4], Yong K Zhu[1,2,3], Jennifer A. Doudna[1,2,3,4,5,6,7]

[1]Gladstone-UCSF Institute of Data Science and Biotechnology; San Francisco, CA, USA

[2]Department of Molecular and Cell Biology, University of California, Berkeley; Berkeley, CA, USA

[3]Innovative Genomics Institute; University of California, Berkeley, CA, USA

[4]California Institute for Quantitative Biosciences, University of California, Berkeley; Berkeley, CA, USA.

[5]Howard Hughes Medical Institute, University of California, Berkeley; Berkeley CA, USA

[6]Molecular Biophysics and Integrated Bioimaging Division, Lawrence Berkeley National Laboratory; Berkeley, CA, USA.

[7]Department of Chemistry, University of California, Berkeley; Berkeley, CA, USA.

*Corresponding author: doudna@berkeley.edu

Supplementary Fig. 1 - Uncropped blot and TLC images

Fig 4E

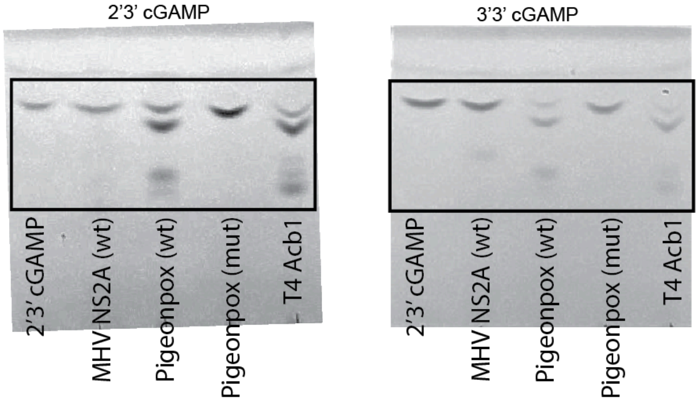

Supplementary Fig. 9A

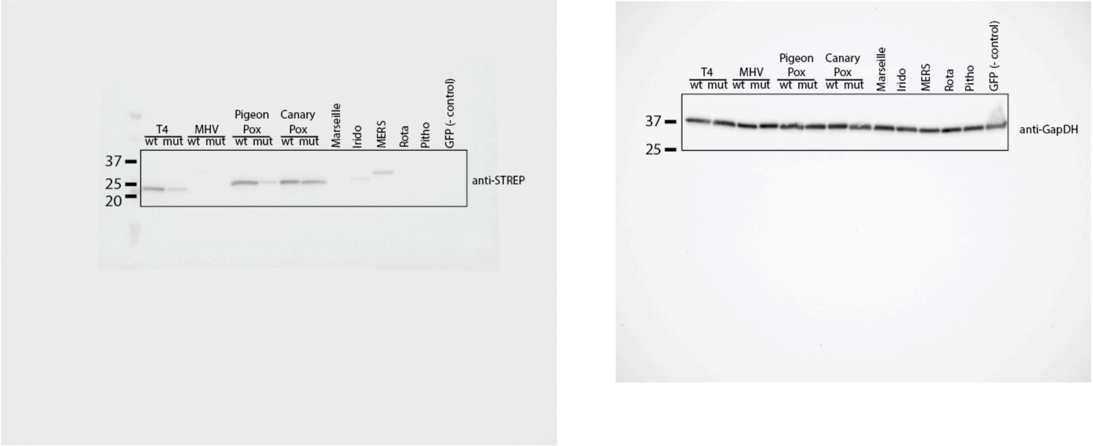

Supplementary Fig. 9C

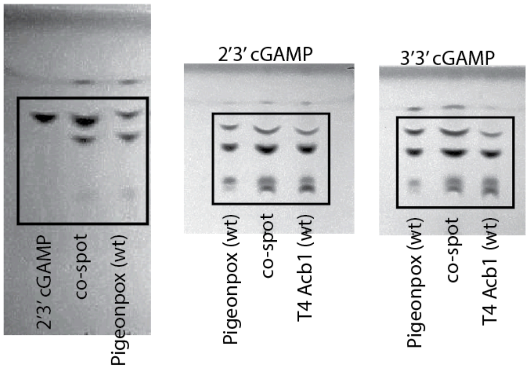

Supplementary Fig 1. - Uncropped blot and TLC images for Fig 4E and Supplementary Figs 9A and 9C.
