# Supplementary material for: Birth of protein folds and functions in the virome
Source: Nature. 2024 Aug 26;633(8030):710–7. doi: 10.1038/s41586-024-07809-y (PMC11410667; doi:10.1038/s41586-024-07809-y)
Supplement: Supplementary file 2 — Reporting Summary [file 41586_2024_7809_MOESM2_ESM.pdf]

nature portfolio

Corresponding author(s): Jennifer Doudna

Last updated by author(s): Jan 10, 2024

Reporting Summary

Nature Portfolio wishes to improve the reproducibility of the work that we publish. This form provides structure for consistency and transparency in reporting. For further information on Nature Portfolio policies, see our Editorial Policies and the Editorial Policy Checklist.

Statistics

For all statistical analyses, confirm that the following items are present in the figure legend, table legend, main text, or Methods section.

n/a	Confirmed	
☐	☒	The exact sample size (*n*) for each experimental group/condition, given as a discrete number and unit of measurement
☐	☒	A statement on whether measurements were taken from distinct samples or whether the same sample was measured repeatedly
☐	☒	The statistical test(s) used AND whether they are one- or two-sided<br>*Only common tests should be described solely by name; describe more complex techniques in the Methods section.*
☒	☐	A description of all covariates tested
☒	☐	A description of any assumptions or corrections, such as tests of normality and adjustment for multiple comparisons
☐	☒	A full description of the statistical parameters including central tendency (e.g. means) or other basic estimates (e.g. regression coefficient) AND variation (e.g. standard deviation) or associated estimates of uncertainty (e.g. confidence intervals)
☐	☒	For null hypothesis testing, the test statistic (e.g. *F*, *t*, *r*) with confidence intervals, effect sizes, degrees of freedom and *P* value noted<br>*Give P values as exact values whenever suitable.*
☒	☐	For Bayesian analysis, information on the choice of priors and Markov chain Monte Carlo settings
☒	☐	For hierarchical and complex designs, identification of the appropriate level for tests and full reporting of outcomes
☐	☒	Estimates of effect sizes (e.g. Cohen's *d*, Pearson's *r*), indicating how they were calculated

Our web collection on statistics for biologists contains articles on many of the points above.

Software and code

Policy information about availability of computer code

Data collection

Code used for upstream processing is present in the vpSAT Github repository (https://github.com/jnoms/vpSAT/tree/main). This includes scripts required for most computational steps. A workflow is available that shows all main processing steps: https://github.com/jnoms/vpSAT/blob/main/manuscript_code/latest/analysis_workflow.ipynb. The stable vpSAT version used for this work is available through zenodo: https://zenodo.org/doi/10.5281/zenodo.10373132.

The SAT python package can be downloaded as instructed on the SAT Github repository (https://github.com/jnoms/SAT/tree/main). The stable SAT version used for this work is available through zenodo: https://zenodo.org/doi/10.5281/zenodo.10373132.

Code used for phylogenetics analysis can be found here: https://github.com/Doudna-lab/nomburg_j-LigT_phylogeny.

All plotting and analysis scripts are available as Quarto documents: https://github.com/jnoms/vpSAT/blob/main/manuscript_code/2024-01-04/.

All code can also be found on Zenodo, along with all intermediate data necessary to reproduce the figures: https://zenodo.org/doi/10.5281/zenodo.10373132

Other software versions:
MMseqs2 release version b0b8e85f3b8437c10a666e3ea35c78c0ad0d7ec2
Colabfold downloaded June 22, 2023
Dalilite version 5
Foldseek version 2.8bd520

IQTree version 2.3.3
Clustal Omega v1.2.4
MMseqs2 version 15.6f452 (for the phylogenetics analysis)
InterProScan version 5
PSI-BLAST version 2.15.0
DIAMOND version 0.9.14
jackhmmer version 3.1b2
hhsuite version 3.3.0

Data analysis	R version 4.0.3 - all analysis code is available in the Zenodo repository

For manuscripts utilizing custom algorithms or software that are central to the research but not yet described in published literature, software must be made available to editors and reviewers. We strongly encourage code deposition in a community repository (e.g. GitHub). See the Nature Portfolio guidelines for submitting code & software for further information.

Data

Policy information about availability of data

All manuscripts must include a data availability statement. This statement should provide the following information, where applicable:

- Accession codes, unique identifiers, or web links for publicly available datasets
- A description of any restrictions on data availability
- For clinical datasets or third party data, please ensure that the statement adheres to our policy

There are several options for viewing, downloading, and searching the structural models generated here.

Searching
We have established a Google Colab notebook that enables any user to quickly and easily search one or more protein structures against our viral structure database using Foldseek: https://colab.research.google.com/github/jnoms/vpSAT/blob/main/bin/colab/QueryStructures.ipynb . This notebook runs rapidly and displays alignment results and information on the protein clusters to which alignment targets belong.
For users who want to conduct high-throughput searches, we have released a pre-made Foldseek database to facilitate use - https://zenodo.org/doi/10.5281/zenodo.10685504.

Viewing and downloading
We have established a Google Colab notebook that allows users to explore our data. Users can input a virus taxonomy ID or family name and browse available proteins. Users can then automatically view and download individual structures. https://colab.research.google.com/github/jnoms/vpSAT/blob/main/bin/colab/ExploreStructures.ipynb .
We have uploaded our structures to ModelArchive - https://www.modelarchive.org/doi/10.5452/ma-jd-viral. ModelArchive hosts predicted structures in a uniform way with extensive metadata. Furthermore, ModelArchive is part of EBI's 3D-Beacons framework (https://www.ebi.ac.uk/pdbe/pdbe-kb/3dbeacons/), enabling uniform downloads and processing of our protein structures through a shared API encompassing the PDB, Alphafold database, and other databases.
Structures can be accessed through each viral family phage in Viralzone [ref] - https://viralzone.expasy.org/10977.
Finally, all structures are available on Zenodo: https://zenodo.org/doi/10.5281/zenodo.10291580

Research involving human participants, their data, or biological material

Policy information about studies with human participants or human data. See also policy information about sex, gender (identity/presentation), and sexual orientation and race, ethnicity and racism.

Reporting on sex and gender	N/A
Reporting on race, ethnicity, or other socially relevant groupings	N/A
Population characteristics	N/A
Recruitment	N/A
Ethics oversight	N/A

Note that full information on the approval of the study protocol must also be provided in the manuscript.

Field-specific reporting

Please select the one below that is the best fit for your research. If you are not sure, read the appropriate sections before making your selection.

☒ Life sciences ☐ Behavioural & social sciences ☐ Ecological, evolutionary & environmental sciences

For a reference copy of the document with all sections, see nature.com/documents/nr-reporting-summary-flat.pdf

Life sciences study design

All studies must disclose on these points even when the disclosure is negative.

Sample size	There was no consideration of sample size. We simply included all viral proteins available from RefSeq at the start of this study.
Data exclusions	There were two key exclusions:<br>1. Proteins that were larger than 1500 residues (or, in some cases 1000 residues) were excluded. This resulted in exclusion of 1706 proteins.<br>2. A small number of proteins failed to fold.
Replication	All computational pipelines were run successfully at least twice.
Randomization	This study did not perform experiments that require sample randomization.
Blinding	This study did not perform experiments that require blinding.

Reporting for specific materials, systems and methods

We require information from authors about some types of materials, experimental systems and methods used in many studies. Here, indicate whether each material, system or method listed is relevant to your study. If you are not sure if a list item applies to your research, read the appropriate section before selecting a response.

Materials & experimental systems

n/a	Involved in the study	
☐	☒	Antibodies
☐	☒	Eukaryotic cell lines
☒	☐	Palaeontology and archaeology
☒	☐	Animals and other organisms
☒	☐	Clinical data
☒	☐	Dual use research of concern
☒	☐	Plants

Methods

n/a	Involved in the study	
☒	☐	ChIP-seq
☒	☐	Flow cytometry
☒	☐	MRI-based neuroimaging

Antibodies

Antibodies used	Streptactin HRP (IBA 2-1502-001), Santa Cruz Biotech Mouse anti GapDH (sc-365062), ECL Anti-mouse IgG (Amersham NXA931)
Validation	These antibodies are reported to be validated by their manufacturers.

Eukaryotic cell lines

Policy information about cell lines and Sex and Gender in Research

Cell line source(s)	HEK 293T cells were kindly provided by the Ott lab at the Gladstone Institutes. These cells were originally provided by ATCC.
Authentication	None of the cell lines were authenticated.
Mycoplasma contamination	Cells were tested for Mycoplasma by the Gladstone Institutes Stem Cell Core within the past year, and determined to be Mycoplasma free.
Commonly misidentified lines<br>(See ICLAC register)	No commonly misidentified cell lines were used in this study.

Plants

Seed stocks

Report on the source of all seed stocks or other plant material used. If applicable, state the seed stock centre and catalogue number. If plant specimens were collected from the field, describe the collection location, date and sampling procedures.

Novel plant genotypes

Describe the methods by which all novel plant genotypes were produced. This includes those generated by transgenic approaches, gene editing, chemical/radiation-based mutagenesis and hybridization. For transgenic lines, describe the transformation method, the number of independent lines analyzed and the generation upon which experiments were performed. For gene-edited lines, describe the editor used, the endogenous sequence targeted for editing, the targeting guide RNA sequence (if applicable) and how the editor was applied.

Authentication

Describe any authentication procedures for each seed stock used or novel genotype generated. Describe any experiments used to assess the effect of a mutation and, where applicable, how potential secondary effects (e.g. second site T-DNA insertions, mosiacism, off-target gene editing) were examined.
